# Lack of evidence for the efficacy of enhanced surveillance compared to other specific interventions to control neonatal healthcare-associated infection outbreaks

**DOI:** 10.1093/trstmh/trv116

**Published:** 2016-01-28

**Authors:** J. Birt, K. Le Doare, C. Kortsalioudaki, J. Lawn, P. T. Heath, M. Sharland

**Affiliations:** a Public Health England, Manor Farm Road, Porton Down SP4 0JG, UK; b Manchester University, Oxford Rd, Manchester M13 9PL, UK; c St George's University of London, Blackshaw Road, London SW17 0TE, UK; d Centre for International Child Health, Imperial College London, Norfolk Place, London W2 1PG, UK; e London School of Hygiene and Tropical Medicine, Keppel St, London WC1E 7HT, UK

**Keywords:** Hospital-acquired infection, Neonates, Outbreaks, Prevention

## Abstract

**Background:**

Despite current prevention efforts, outbreaks of healthcare-associated infections in neonatal units remain high globally, with a considerable burden of mortality and morbidity.

**Methods:**

We searched Medline, Cochrane Library and Outbreak database to identify studies of neonatal healthcare-associated outbreaks between 2005 and 2015 that described interventions to control outbreaks. All studies were evaluated using the ORION guidance.

**Results:**

Thirty studies were identified including 17 102 infants of whom 664 (3.9%) became infected. No single intervention was identified that reduced duration or mortality. Studies that introduced multiple interventions had significantly reduced case fatality ratio and outbreak duration compared to those that used basic surveillance only. Low and low-middle income countries reported the fewest interventions to control outbreaks and these studies were also associated with higher mortality than that found in middle and high income countries.

**Conclusions:**

Systematic reporting and formal evaluation of interventions used to reduce healthcare-associated neonatal infection outbreaks is key to identifying containment strategies worldwide.

## Introduction

The reported incidence of outbreaks on neonatal units has increased in the last 20 years from 5.7 outbreaks per year in the 1990s to 10.1 outbreaks per year in the 2000s.^1^ In the published literature there is little differentiation made between hospital acquired infection (HAI) prevention strategies and outbreak control practices and interpretation of studies reporting outbreaks is often limited by methodological weaknesses such as lack of details on study design and the nature and timing of interventions, as well as failure to identify potential sources of bias within the studies.^2^

There are important differences between outbreaks on neonatal intensive care units (NICU) and those in other hospital departments that may affect the efficacy of interventions to terminate an outbreak (Box 1). Compared to adult intensive therapy units (ITU), NICU outbreaks are most commonly associated with Gram-negative pathogens with high rates of antimicrobial resistance.^3^ Additionally, outbreaks in NICU involve a high patient burden (average of 23.9 patients vs 6.9 in adult ITUs). Finally, in contrast to adult ITU outbreaks, approximately 50% of the published NICU outbreaks had no source identified.^1^

Box 1. **Differences between NICU and PICU or adult ITU**

- Longer median length of stay compared to PICU or ITU
- Infant immature immune system and related immune deficiency
- Immature infant gut may lead to prolonged colonisation with disease-causing pathogens
- Poor skin barrier further increases infection risk

ITU: intensive therapy unit; NICU: neonatal intensive care unit; PICU: pediatric intensive care unit

The aim of this systematic review was to determine the effect of reported outbreak control initiatives on the duration and case fatality ratio (CFR) of outbreaks in neonatal units globally based on published studies of outbreaks using the ORION guidelines.^1^

## Methods

### Search strategy and selection criteria

We searched Medline, Cochrane Library and the Outbreaks database (www.outbreak-database.com) to identify studies of neonatal healthcare-associated outbreaks globally that described the specific interventions used to control the outbreak, confined to bacterial studies. We searched for reports published between 1 January 2005 and 30 June 2015, with no language restriction. We used a comprehensive list of terms; for Medline/Cochrane Library: infant OR neonate [MeSH Terms] AND (infection OR sepsis OR bacteremia [MeSH Terms] OR cross infection, infection control, disease outbreaks [MeSH Terms] OR (disease [All Fields] AND outbreaks [All Fields] OR disease outbreaks [All Fields] OR outbreak [All Fields] OR nosocomial [All Fields] AND infection [All Fields] OR nosocomial infection [All Fields]), published between 2005 and 2015; for the Outbreak database: all reported outbreaks reported between 1 January 2005 and 31 May 2015.

Two authors (JB and CK) screened abstracts of retrieved references for potentially relevant studies containing data on healthcare-associated bacterial outbreaks on a neonatal unit, describing the interventions used for outbreak control, timing of intervention to end of outbreak and CFR. When we found duplicate reports of the same study in preliminary abstracts and articles, we analyzed data from the most complete data set. Included studies were required to have identified the methods used for identifying the bacteria, and describe interventions used to isolate the cause and manage the outbreak. All articles were required to quantify the total number of inpatients during the outbreak, number of cultures obtained, number of infected patients and details of patient gestational age and gender. Only studies meeting our minimum requirements for data completeness described above were included.

We excluded community-acquired infections, outbreaks describing colonization but no disease, and outbreaks on adult and pediatric intensive care units or mixed outbreaks where data was not described separately. We obtained the full text of potentially relevant studies and two authors (JB and CK) scrutinized these reports independently. We also screened reference lists of all reviewed studies for further eligible publications.

### Data extraction

Extracted data included: authors; year of publication; countries of study; timing of outbreak; setting and scope of study; sample size; surveillance methods; definitions used for diagnosis; reported infection prevalence or cumulative incidence data and corresponding denominators; microbiological isolates; antimicrobial resistance, interventions used and methods used to assess efficacy of these interventions. We further stratified studies according to World Bank classification as high income, upper-middle income, lower-middle income and low income countries.^4^ After extraction, data were reviewed and compared by the third author (KLD). Instances of disagreement between the three extractors were solved by a consensus among the investigators. Whenever needed, we obtained additional information about a specific study by directly questioning the principal investigator. The interventions were allocated to groups: basic surveillance (defined as environmental swabs and monitoring of results) only, enhanced surveillance level 1a: basic surveillance + cohorting; enhanced surveillance level 1b: basic surveillance, cohorting + cleaning; enhanced surveillance level 2: basic surveillance, cohorting, cleaning + infection control team surveillance; enhanced surveillance level 3: basic surveillance, cohorting, cleaning, infection control team surveillance + care bundles and enhanced surveillance level 4: basic surveillance, cohorting, cleaning, infection control team, care bundles + closure of facility.

### Validity assessment

We used the PRISMA guidelines^2^ to report studies and the ORION^1^ approach to summarize the quality of evidence for each outcome.^2^ Study type, quality, limitations, imprecision, inconsistency, indirectness, or suspicion of a reporting bias are assessed. We used the GRADE system to assess the quality of articles.^5^ Quality assessment was done by two reviewers for all studies (JB, KLD). Quality of studies was graded as very low (0), low (1), medium (2) or high (3).

### Definitions

Neonatal studies were defined as patients less than 28 days of age on an NICU. Standardized definitions (i.e., according to the US Centers for Disease Control and Prevention National Nosocomial Infection Surveillance [NNIS]/National Healthcare Safety Network [NHSN] system) for hospital-associated outbreaks were used throughout. Case fatality ratio was defined as the proportion of reported cases which are fatal during the study period.

### Statistical analysis

We pooled data from outbreak studies by pathogen and region and summarized the results. All data was weighted according to number of patients screened. Using a random effects meta-analysis we calculated mean and median outbreak duration and CFR by pathogen by country and World Bank classification. Additionally we calculated the time from recognition of outbreak to final negative culture. We describe the interventions used for each outbreak using a weighted mean. We were unable to conduct meta-analysis or multivariate analysis by pathogen or country classification by intervention due to the small number of studies in each group.

## Results

### Characteristics of the studies

**Figure 1.**
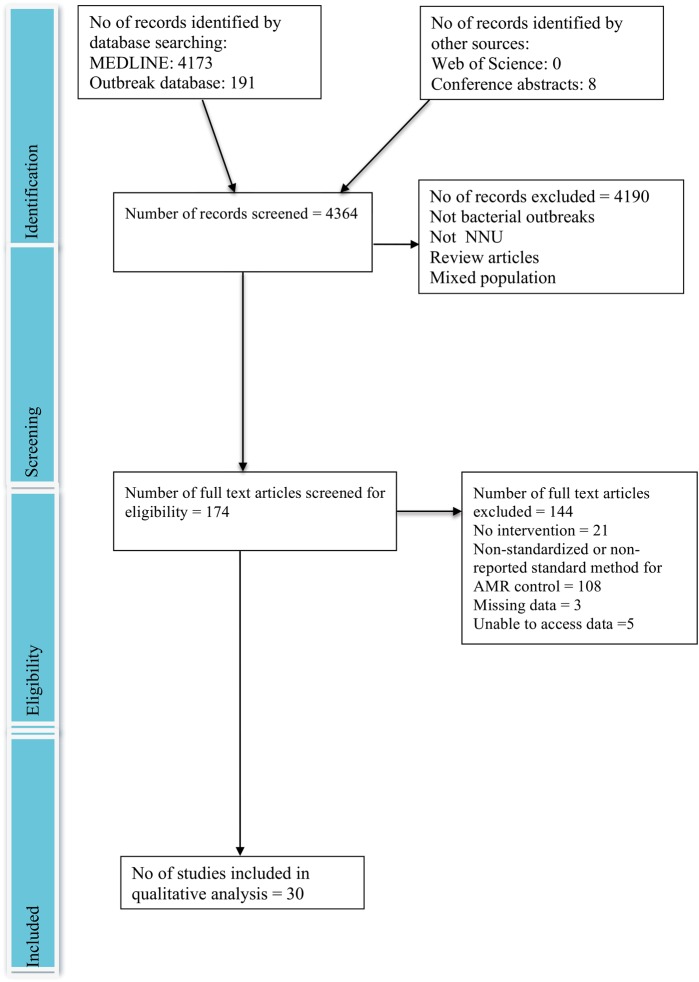
Flowchart of study selection process (based on PRISMA flowchart).^2^ This figure is available in black and white in print and in color at Transactions online.

The online database search performed on 1 July 2015 yielded 4364 articles (Figure 1). Thirty selected articles representing unique patient cohorts,^3–32^ were eligible for systematic review. The eligible studies were undertaken in 30 NICUs in 15 locations. Nineteen (63%) came from high income countries,^3–21^ four (13%) from upper-middle income countries,^22–25^ six (20%) from lower-middle income countries^26–31^ and one (3%) from a low income country.^32^ Six studies reported Methicillin-resistant *Staphylococcus aureus* (MRSA).^3,5,8,12,14,17^ The median age of infants involved in outbreaks was 12 days (3–28).

### Duration of outbreaks

**Figure 2.**
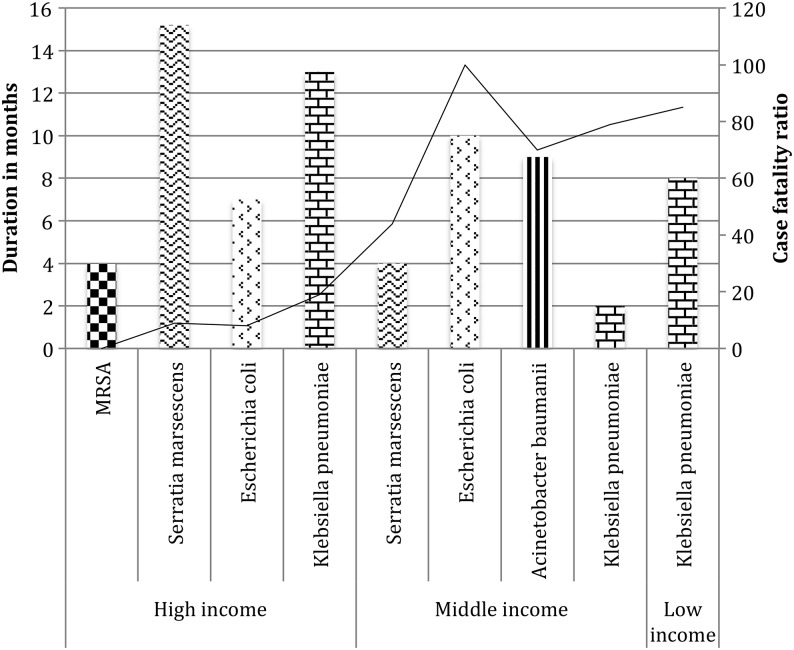
Outbreak duration (months) and (%) case fatality ratio by pathogen by country classification.

Figure 2 and Table 1 show that the duration of the outbreaks studied ranged from 1–22 months (mean 10.4; CI 7.4–13.4; heterogeneity (I2) 97.1%). The mean duration of outbreaks was longest for high-income countries (12 months; CI 8.4–15.7; I2 97.7) compared to lower middle-income countries (2.7 months; 1.5–3.8; I^2^ 94.4). However, longitudinal follow up was longer in high income countries (44.1 months; 40.4–47.7) compared to lower-middle income countries (4.6 months; 3.4–5.8), which may account for this difference (p<0.0001).

**Table 1.** Mean duration and case fatality ratio by pathogen by World Bank classification

Pathogen	No. studies	Bacteria	Outbreak duration (months)	CFR%
		World Bank classification	Mean (95% CI)	Mean (95% CI)
Total all countries	6	MRSA	3.5 (2–8)	0 (0–10)
	6	*Serratia marcescens*	14.6 (8.9–20.4)	11 (4–19)
	2	*Escherichia coli*	7 (2.5–11.5)	8 (0–23)
	10	*Klebsiella pneumoniae*	6.5 (5.4–7.5)	49 (41–57)
	2	*Acinetobacter baumanii*	9 (6.8–11.2)	67 (59–75)
	4	Other	8.5 (3–11.5)	17 (5–26)
		World Bank classification		
MRSA	6	High	3.5 (2–8)	0 (0–10)
		Upper–middle	NA	NA
		Lower–middle	NA	NA
		Low	NA	NA
*S. marcescens*	5	High	15.4 (9.3–21.5)	9 (1–17)
		Upper–middle	NA	NA
	1	Lower–middle	4	44
		Low	NA	NA
*E. coli*	1	High	7	7
		Upper–middle	NA	NA
	1	Lower–middle	10	100
		Low	NA	NA
*K. pneumoniae*	5	High	12.8 (10.9–14.7)	17 (4–29)
	1	Upper–middle	1	0
	3	Lower–middle	2.2 (0.9–3.6)	79 (64–87)
	1	Low	8	85
*A. baumanii*		High	NA	NA
	2	Upper–middle	9 (6.8–11.2)	67 (59–75)
		Lower–middle	NA	NA
		Low	NA	NA

CFR: case fatality ratio; MRSA: multi-resistant staphylococcus aureus; NA: not available.

### Pathogens causing outbreaks

The number of infants exposed to outbreak pathogens was 17 102, of whom 664 (3.9%) became infected with an outbreak organism. This varied by pathogen with high transmission rates reported for *Acinetobacter baumanni* (34.1%) and *Klebsiella pneumoniae* (8.1%) in high or upper-middle income countries. Transmission rates of all pathogens were higher in low-middle income countries compared to high/upper-middle income countries (transmission rates: *K. pneumoniae* 33.2% vs 8.1%; *Escherichia coli* 20% vs 0.4%; *Serratia marcescens* 51% vs 20.1%). Outbreaks of *K. pneumoniae* were most commonly reported (33%). All *K. pneumoniae* isolates (28.2%) were extended spectrum β-lactamase (ESBL) producers and where genetic analysis was available, a high prevalence of CTX, NDM, SHV and TEM resistance mutations were identified. MRSA was identified in 20%, all from high-income countries (78/664 infections; 11.8%). *S. marcescens* was also reported in 20%. These isolates were all identified as ESBL-producers (230/664 infections; 34.6%). *A. baumanii* 7% studies; 14.3% isolates) and *E. coli* 7% studies; 3.5% isolates) were infrequently identified as the cause of outbreaks (Table 1).

### Source of the outbreaks

The source of the outbreak was identified in 17 studies: a patient transferred in from another hospital (6/17)^6,7,23–25,29^; healthcare worker (4/17)^13,19,28,30^; contaminated ventilator equipment (5/17)^9,16,21,31^ and mothers colonized on surface swabs (2/17).^4,26^

### Case fatality ratio

The CFR across all studies was 20% (14–26; I^2^ 95.3%). The CFR varied according to income level with high-income countries reporting lowest CFR (9%, 2–16), whereas upper-middle and lower-middle countries reported higher mean CFR at 53% (46–61) and 70% (61–79) respectively. Only one paper was from a low-income level country and this reported a fatality ratio of 22%.^32^ The CFR also varied according to pathogen with *A. baumanii* 67% (59–75) and *K. pneumoniae* 49% (41–57) showing higher mortality compared to MRSA (0%) (Figure 2).

### Interventions

#### Basic surveillance plus hand hygiene strategies, patient and environmental screening (enhanced basic intervention)

In addition to surveillance of bacterial cultures, all studies implemented stringent hand hygiene strategies (including education and monitoring) and enhanced patient and environmental screening strategies at the start of the outbreak (Table 2). However, the quality of the evidence of effectiveness was graded as low as these results were from retrospective cohort studies with small patient numbers.

**Table 2.** Mean and median duration and CFR of Gram negative bacteria outbreaks by number of interventions and World Bank country classification^4^

No. interventions	n=20	Intervention	Duration months		CFR %	
			Mean (CI)	Median (IQR)	Mean (CI)	Median (IQR)
Total	2	Basic intervention (A)	8 (5.2–10.8)	7 (7–8)	54 (7–100)	54 (7–100)
	2	Enhanced surveillance level 1a: A + cohorting (B)	6.5 (0–12)	4 (4–6.5)	16 (0–32)	16 (0–32)
	4	Enhanced surveillance level 1b: B + cleaning (C)	10.8 (0–25.1)	5.5 (4–8.5)	40 (8–72)	39 (27–54)
	4	Enhanced surveillance level 2: C + infection control team surveillance (D)	7.8 (2–12.8)	6 (4–8.5)	39 (0–100)	33 (15–63)
	6	Enhanced surveillance level 3: D + care bundles (E)	4.5 (0–15.9)	1 (1–1.5)	30 (0–100)	23 (10–50)
	2	Enhanced surveillance level 4: E + closure of facility	9.5 (9–11)	9 (9–9.5)	35 (7–63)	35 (25–45)
High income	1	Basic intervention (A)	7	7	7	7
	0	Enhanced surveillance level 1a: A + cohorting (B)	NA	NA	NA	NA
	3	Enhanced surveillance level 1b: B + cleaning (C)	12 (0–30)	4 (4–10)	35 (7–63)	29 (25–50)
	1	Enhanced surveillance level 2: C + infection control team surveillance (D)	10	10	8	8
	4	Enhanced surveillance level 3: D + care bundles (E)	6.3 (0–18)	1.5 (1–4)	12 (0–48)	3 (0–23)
	1	Enhanced surveillance level 4: E + closure of facility	9	9	45	45
Upper-middle	0	Basic intervention (A)	NA	NA	NA	NA
	2	Enhanced surveillance level 1a: A + cohorting (B)	6.5 (0–12)	4 (4–6.5)	16 (0–64)	16 (0–32)
	0	Enhanced surveillance level 1b: B + cleaning (C)	NA	NA	NA	NA
	1	Enhanced surveillance level 2: C + infection control team surveillance (D)	9	9	83	83
	0	Enhanced surveillance level 3: D + care bundles (E)	NA	NA	NA	NA
	0	Enhanced surveillance level 4: E + closure of facility	NA	NA	NA	NA
Lower-middle	1	Basic intervention (A)	9	9	100	100
	0	Enhanced surveillance level 1a: A + cohorting (B)	NA	NA	NA	NA
	1	Enhanced surveillance level 1b: B + cleaning (C)	7	7	57	57
	1	Enhanced surveillance level 2: C + infection control team surveillance (D)	4	4	44	44
	2	Enhanced surveillance level 3: D + care bundles (E)	1	1	67 (20–100)	67 (50–85)
	1	Enhanced surveillance level 4: E + closure of facilities	10	10	25	25
Low	0	Basic intervention (A)	NA	NA	NA	NA
	0	Enhanced surveillance level 1a: A + cohorting (B)	NA	NA	NA	NA
	0	Enhanced surveillance level 1b: B + cleaning (C)	NA	NA	NA	NA
	1	Enhanced surveillance level 2: C + infection control team surveillance (D)	8	8	22	22
	0	Enhanced surveillance level 3: D + care bundles (E)	NA	NA	NA	NA
	0	Enhanced surveillance level 4: E + closure of facility	NA	NA	NA	NA

CFR: case fatality ratio; NA: not available.

(A) = basic surveillance; (B) = (A) + cohorting; (C) = (B) + cleaning; (D) = (C) + infection control team; (E) = (D) + care bundles.

#### Enhanced intervention level 1: cohorting and enhanced cleaning

Two studies^14,32^ added cohorting to enhanced basic intervention. In both studies outbreak duration and mortality were reduced compared to basic intervention only (median duration 4 months [4–6.5] vs 7 months [7–8]; p=0.03); median CFR 16% (0–32) vs 54% (7–100); p=0.01). It was not possible to analyze the addition of cohorting by pathogen as study numbers were too small. Cohorting combined with equipment and environmental deep cleaning were added to basic intervention in four studies.^21,23,24,28^ The addition of cleaning and cohorting reduced the reported outbreak duration compared to basic intervention strategies (5.5 months [4–8.5] vs 7 months [7–8]; p=0.04; CFR 39 [27–54] vs 54 [7–100]; p=0.02). The low number of studies outside of the high-income setting precluded analysis by country index.

The quality of the evidence was graded as moderate as these results were from prospective cohort studies with large patient numbers.

#### Enhanced intervention level 2: developing a dedicated outbreak infection control team

Four studies reported the development of a dedicated infection control team to monitor the outbreak, conduct audits of hygienic practices and educate staff.^6,7,19,31^ Compared to enhanced basic intervention only, CFR was reduced (33% [15–63] vs 54% [7–100]) but not outbreak duration (6 months [4–8.5] vs 7 months [7–8]). It was not possible to compare by country index or pathogen.

The quality of the evidence was graded as low as these results were from retrospective cohort studies with small patient numbers.

#### Enhanced intervention level 3: introduction of care bundles

Six studies added care bundles to basic intervention, together with cleaning, cohorting and the development of an infection control team.^4,10,11,26,27,29^ The bundles included catheter and ventilator care bundles, guidance and education on catheter and ventilator care, daily audit of practices, anonymous reporting of breaches to protocols, staff education, deep clean protocols and antimicrobial stewardship. Compared to basic intervention only, the reported outbreak duration was considerably shorter 1 month (1–1.5) vs 7 months (7–8) and CFR was lower (23%, [10–50] vs 54%, [7–100]). This was true for high-income countries (1.5 months [1–4] vs 4 [4–10] for enhanced intervention level 1); CFR 3% (0–23) vs 29% (25–50). The CFR, but not outbreak duration, was reduced if care bundles were added to other interventions in high income countries (15%, [8–22] vs 25%, [0–50]). There were insufficient studies in other country areas or by specific pathogens to be able to undertake further analysis. The quality of the evidence was graded as moderate as these results were from large, prospective cohort studies.

#### Step-wise introduction of interventions

Three studies describe a stepwise introduction to outbreak control interventions.^13,20,29^ All described step one as basic intervention, which failed to stop the outbreak in any unit. During the second phase of interventions, staff education of hygienic practices and the initiation of alcohol-based hand gel at each incubator were implemented. The authors of all three studies indicated that a combination of education and hand gel availability was responsible for the cessation of the outbreaks on these units. However, all three studies reported further outbreaks from prolonged surveillance.

### Follow up at the end of the outbreak

An enhanced antimicrobial stewardship policy was instigated in 7/10 high-income countries but only in 1/3 upper-middle income and 1/6 low-middle income countries. Guidelines for NICU cleaning and hygienic practices were changed in 12/30 studies. This included: 6/10 high income, 1/3 upper-middle income, 4/6 low-middle income and 1/1 low income countries.

### Re-evaluation of interventions following the end of outbreak

Point-prevalence rectal swabs and/or environmental swabs were undertaken in 15/20 studies to assess the recurrence of Gram-negative pathogens.^4,6–9,13,16,18,20,21,23–25,28,29^ Four studies reported subsequent new outbreaks despite changes to guidelines and education.^11,20,21,27^ The remaining studies did not identify any further outbreaks, although the follow up period varied from 1–50 months.

## Discussion

### Main findings

In this systematic review we highlight that outbreaks on neonatal units represent a major burden and a serious patient safety issue for hospitalized neonates in both high and low resource settings. Our finding that enhanced surveillance (surveillance of environmental and patient cultures and hand hygiene) appears to be relatively ineffective at reducing either case fatality ratios or outbreak duration has important implications for hospital infection control practices. The evidence suggests that a stepwise bundle approach to outbreak prevention should include: equipment care, ongoing staff education and training, regular audit of practices and reporting of protocol breeches, deep clean protocols and antimicrobial stewardship as the most effective approach to reducing case fatality ratio and duration of outbreak globally (Box 2).

Box 2. **Recommendations for a stepwise bundle approach to outbreak management**

- Hand-hygiene practices
- Equipment care
- Ongoing staff education and training
- Regular audit of practices and reporting of protocol breeches
- Deep clean protocols
- Antimicrobial stewardship

The burden of HAI outbreaks in neonatal units is especially high in low-middle income settings where CFR rates were reported to be between 64 and 100%. Neonates admitted to newborn nurseries in low and middle income countries are often more at risk from healthcare associated infections because of less hygienic care practices and more limited environmental cleaning.^36^ Commonly identified sources of outbreaks include contaminated intravenous solutions, tubing and supplies as well as environmental surfaces (e.g., drug preparation surfaces or cots), or the colonized hands of staff who handle neonates.^37^ The high rates of neonatal infection and the type of pathogen commonly identified strongly suggest that lack of appropriate hygiene during labor, delivery and in the early postnatal period are major contributors to infant mortality and morbidity due to infection.^37,38^

### Comparison to other studies

Outbreaks in neonatal units have been widely studied. However, despite the large number of studies identified, few rigorously described the outbreak in such a way that the efficacy of interventions could be adequately assessed. Whilst practical interventions to reduce the burden of hospital-associated infections have been developed for adults,^39^ there is little evidence of the effectiveness of these practices on NICUs where outbreaks are often associated with a lack of specific infection control practices.^37^ Absence of clean water and soap for handwashing is often noted during outbreaks in resource-poor settings.^38^ Alcohol-based antiseptics for hand hygiene are an appealing innovation because they reduce hand contamination and are easy to use, especially when clean water is not available. However, there are no randomized control trials of efficacy in neonatal outbreak settings and these antiseptics are expensive. Two studies conducted prior to this review suggest that use of hand gel does reduce late onset neonatal infections^40,41^ and evidence from adult studies is encouraging.^39^ Anecdotal evidence from two studies in this review also indicate that the provision of hand gel many have reduced outbreak duration, although both were small cohort studies and hand hygiene practices alone had little impact on either CFR or duration of the outbreak.

Several studies have identified staff education and engagement as key to the success of managing hospital-associated infections.^42^ Staff education and audit is a recognized tool for the implementation of infection control programmes in both adult and pediatric units.^43–45^ However, initiatives are difficult to implement and without staff engagement are difficult to sustain.^46–48^ A recent review of global outbreaks identified understaffing as a major risk factor for outbreaks of ESBL-infections in NICUs but failed to identify interventions that would reduce CFR or terminate the outbreak.^49^

In this review, studies that introduced multiple interventions had significantly reduced CFR and outbreak duration compared to those that used basic surveillance only. However, these studies were predominantly from high-income countries and the feasibility of these interventions in resource-poor settings is uncertain. However, a study of bundle interventions in the Philippines where the instigation of alcohol-based hand gel, equipment checking and antimicrobial stewardship reduced CFR by 50% is reassuring^50^; a similar report from Argentina indicated that a combination of hand hygiene and catheter care bundles reduced bacteremia rates.^51^ However, neither these studies nor this review could identify which components of an outbreak intervention bundle would be most beneficial in reducing outbreak duration and mortality. Neonatal nurseries in low and middle income countries are often overcrowded and understaffed with infants sharing cots because of lack of space and equipment. Lack of supplies mean that disinfection may be suboptimal and the addition of these interventions may be difficult in low-resource settings.

### Limitations of the study

This study has limitations. Firstly, we recognize that outbreaks reported in the literature may not be fully representative of all outbreaks as the great majority (and less severe outbreaks) are likely to be unreported.^52^ Although we used the ORION criteria for this systematic review this tool is currently not widely in use for outbreak reporting. Its rigorous approach to grading studies may mean that less robust methodological studies which might be important in generating hypotheses on outbreak containment were not included.^53^ Although we used a wide variety of search criteria, some qualitative studies may have been missed because of less discerning key words. However, using only ‘neonatal’ as a search term in the outbreak database did not reveal further studies of sufficient quality to be included in this review. Studies were only eligible if they we published after 2005 and thus earlier reports of interventions were not included. However, a review by Allegranzi et al. in 2011 on hospital associated infections did not identify any further high quality studies of outbreak reporting or intervention prior to 2005.^52^

The strength of this review is that the studies forming the evidence base represent a rigorous selection from hundreds of papers to reduce methodological bias. The ORION approach was helpful because it consistently identified studies of low quality and with incomplete reporting.

## Conclusions

Our review has demonstrated the marked lack of evidence for neonatal HAI outbreak management globally. We suggest that, based on the limited evidence available, best practice at the onset of an outbreak would include the rapid implementation of hand-hygiene practices, equipment care and deep clean protocols and staff education, and training. An audit reporting mechanism to ensure compliance and antimicrobial stewardship programmes should also be instigated. Randomisation to different levels of enhanced intervention in a clinical trial would provide further insight into the effectiveness of different bundle components. A standardised reporting and intervention tool such as the Outbreak database should be used to report all outbreaks in order to allow for international monitoring and evaluation of the efficacy and cost-effectiveness of these interventions in reducing the burden of outbreaks in NICUs globally.
